# Mining housekeeping genes with a Naive Bayes classifier

**DOI:** 10.1186/1471-2164-7-277

**Published:** 2006-10-30

**Authors:** Luna De Ferrari, Stuart Aitken

**Affiliations:** 1 School of Informatics, the University of Edinburgh, Edinburgh EH8 9LE, UK

## Abstract

**Background:**

Traditionally, housekeeping and tissue specific genes have been classified using direct assay of mRNA presence across different tissues, but these experiments are costly and the results not easy to compare and reproduce.

**Results:**

In this work, a Naive Bayes classifier based only on physical and functional characteristics of genes already available in databases, like exon length and measures of chromatin compactness, has achieved a 97% success rate in classification of human housekeeping genes (93% for mouse and 90% for fruit fly).

**Conclusion:**

The newly obtained lists of housekeeping and tissue specific genes adhere to the expected functions and tissue expression patterns for the two classes. Overall, the classifier shows promise, and in the future additional attributes might be included to improve its discriminating power.

## Background

### Importance of housekeeping genes

Housekeeping (HK) genes are defined as genes constitutively expressed in all tissues to maintain essential cellular functions. Conversely, tissue specific (TS) genes, are defined as genes only or mainly expressed in a specific tissue or organ, and hence responsible for specific functions and development [1]. Gene expression profiling is a key to characterizing normal and diseased biological states.

Many disciplines need to discriminate between housekeeping and tissue specific genes. In Microbiology, housekeeping genes are known to play a role in enhancing virulence of pathogens and they are studied to find potential drug targets [2,3], while slowly diverging housekeeping genes are used in evolutionary studies to discriminate subspecies [4,5]. In Medicine they are studied to discover if genetic diseases linked to housekeeping genes are more likely to affect multiple organs. In Biology and Physiology housekeeping genes are the key to determine the set of basic functions necessary for cellular life or an organ function [1]. Additionally, many quantitative techniques used for diagnosis and research use housekeeping gene expression as a baseline to normalize numerical values and to detect differential expression. Housekeeping genes like Glyceraldehyde-3-phosphate Dehydrogenase (GADPH), beta-actin, or beta-tubulin are used as standards in assay techniques like microarrays, RT-PCR, Northern blotting (for mRNA levels) and Western blotting (for protein levels).

### Direct assay of expression across tissues

Until now, lists of housekeeping genes have been constructed by testing gene expression in different tissues, with the most recent attempts involving microarrays [6-8]. Unfortunately, microarray techniques suffer from certain limitations. Some are intrinsic to the technique, for example, the mRNA extraction by hybridization of the polyA tail on a polyT column causes a loss of mRNA material that remains attached to the column, a detail particularly important for mRNA expressed at extremely low levels. Another problem is the conversion from mRNA to cDNA where the reverse transcription process introduces a bias toward certain sequences. Other limitations might decrease over time, but are still relevant factors today. Cost, for example, is still an important limitation, and has a crucial impact on replicates. Another limit in commercial microarrays is that not all new genomes or gene sequences are immediately available.

In addition, the DNA probes responsible for the specificity of hybridization with a unique gene are sometimes difficult to create. For example, if the probe is too near to the end of the gene sequence, any splicing event will make the probe useless for recognizing different variants of the same gene. Kothapalli found that most probes were, unfortunately, chosen from the gene end in commercial platforms, and so less useful for distinguishing differentially expressed splicing versions [9]. Additionally, the sensitivity of the technique still limits the possibility of reproducing results: comparisons have found a maximum correlation of 70% between genes recognized as expressed by different commercial platforms like Affymetrix ^®^GeneChips and Amersham ™CodeLink [10]. Tan reports an even more modest correlation [11].

Other limitations are becoming evident over time, due to the constantly evolving history of genome sequencing: some published lists of housekeeping genes contain only the gene identifiers valid at the time the experiments were performed, but not the original sequence of the probe. And in general, quoting Brazma: "gene expression data are meaningful only in the context of a detailed description of the conditions under which they where generated" [12]. To overcome these limitations we describe in this article an alternative system based on genes physical characteristics, that will help in identify housekeeping and tissue specific genes without having to test them in a wet lab against all known tissues.

### Physical characteristics of housekeeping genes

Recent articles have demonstrated that tissue specific and housekeeping genes show distinctive physical characteristics of gene length and chromatin compactness. To date, these features have been analysed individually, while in this work we will exploit their additive power in an integrated learning procedure.

#### Gene length

Eisenberg has shown that housekeeping genes tend to be significantly more compact and shorter than tissue specific genes [13]. The average length for introns, exons, 3' UTR (UnTranslated Region) and 5' UTR is shorter for housekeeping genes than for tissue specific genes. Moreover, housekeeping genes tend to have less exons. The theory that underlies these data is that cells are thrifty: "The transcription process is both slow and costly; it takes 50 milliseconds and two ATP molecules approximately to transcribe a nucleotide. This might be expected to provide selective pressure to make genes as short as functionally possible. The more copies of a gene required for the organism, the stronger this pressure should be" [13]. Additional evidence from Castillo-Davis showed that genes with a large number of expressed sequence tags (ESTs) in public libraries have a significantly shorter average intron length than those with fewer ESTs (and hence less expressed mRNAs) [14].

#### Chromatin compactness

The DNA in the nucleus of eukarya is packed in loops and folded over histones to form units called nucleosomes. The DNA structure in the region upstream of a gene is implicated in gene expression regulation, making it more or less easy for transcription factors to attach to or near the promoter and initiate transcription. Ganapathi analyzed the 5' and 3' flanking regions of housekeeping and tissue specific genes for various attributes of chromatin organization [15]. The study showed that putative Scaffold/Matrix Attachment Regions (S/MAR) are more abundant upstream of tissue specific genes as compared to housekeeping genes. S/MAR attach themselves to the nuclear matrix and hence help the formation of chromatin loops. Genes less frequently expressed (tissue specific) appear to have less accessible and more compact DNA in their promoter region, and so more S/MAR sequences. Conversely, some repeats found to be more abundant upstream of housekeeping genes, like Poly(dA-dT) and (CCGNN)n, destabilize the formation of nucleosomes and, leaving the DNA less packaged, are implicated in maintaining constitutive gene expression [16,17].

### The Naive Bayes classifier

To identify an object, in this case a gene, as belonging to a particular class – e.g. housekeeping vs. tissue specific – using computational techniques belongs to the broad field of classification. For the classification to be successful, each class must show some distinct properties or characteristics. None of the characteristics described in the above sections allows by itself a direct classification of a given gene as housekeeping or not. This work, however, tests if multiple features can be combined to create a powerful classifier. The algorithm of choice is the Naive (or simple) Bayes classifier that finds its origins in the Bayesian theory of probability.

The main advantage of Bayesian classifiers is that they are probabilistic models, robust to real data noise and missing values. The Naive Bayes classifier assumes independence of the attributes used in classification but it has been tested on several artificial and real data sets, showing good performances even when strong attribute dependences are present. In addition, the Naive Bayes classifier can outperform other powerful classifiers when the sample size is small. Using the words of Domingos and Pazzani: "In summary, [...] the Bayesian classifier has much broader applicability than previously thought. Since it also has advantages in terms of simplicity, learning speed, classification speed, storage space and incrementality its use should perhaps be considered more often." [18].

This work classifies housekeeping and tissue specific genes on the basis of physical characteristics only, without directly assaying expression in different tissues. It exploits features already available in databases, like exon length and measures of chromatin compactness and combines them into a Naive Bayes classifier to obtain new lists of housekeeping and tissue specific genes in human, mouse and fruit fly.

## Results

### Classifier Evaluation

#### Attributes

A set of attributes was collected and the corresponding attribute values were fed to the classifier for each transcript of all human, mouse and fruit fly genes. Not all attributes, however, are fit for use in a classifier. First, some attributes are clearly not independent and do not provide any additional advantage when evaluated together. For example the number of exons and the number of introns for each transcript: the number of introns – or intervening sequences between exons – is by definition the number of exons minus one. Second, some attributes are not selective enough between the two classes of interest, like the presence of Poly(dA-dT) of 10 or more bp (base pairs). The attributes remaining after this analysis were, for each transcript: 1. cDNA length (entire pre-splicing mRNA length: exons + introns + other untranslated regions), 2. Coding sequence (CDS) length (exons only), 3. Number of exons, 4. Presence of S/MAR in the 5' region, 5. Presence of S/MAR in the 3' region, 6. Presence of Poly(dA-dT) (with length of 18 or more bp) in the 5' region, 7. Presence of (CCGNN)_2–5 _in the 5' region, 8. Percent of GO terms for the gene that match with the housekeeping GO terms list, 9. Percent of GO terms for the gene that match with the tissue specific GO terms list.

#### Short gene length for housekeeping genes

**Figure 1:**
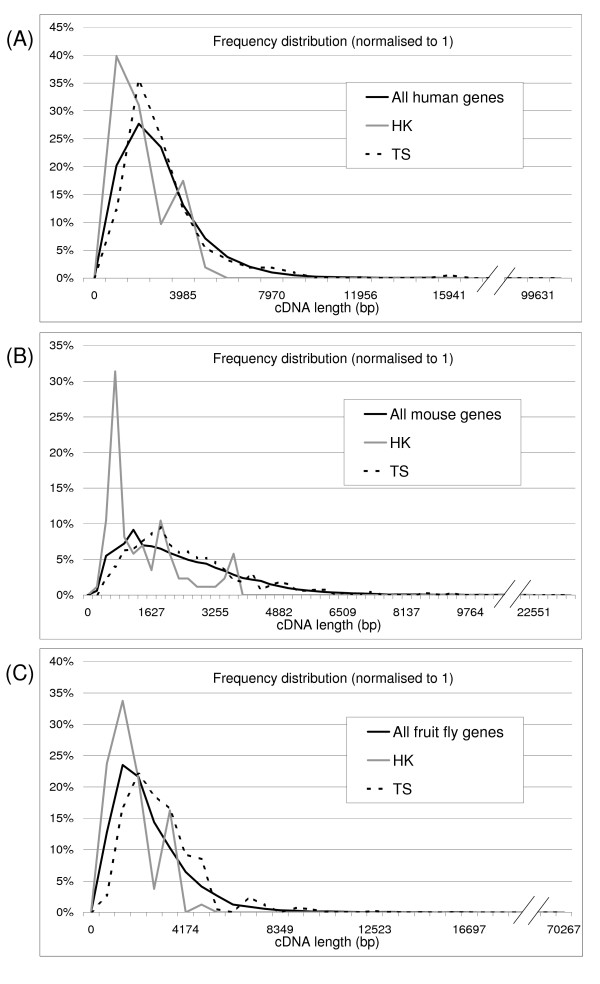
**Frequency distribution of cDNA length in human, mouse and fruit fly transcripts**. The diagrams represent the frequency distribution of cDNA length (normalized to one) for housekeeping, tissue specific and total transcripts in human (A), mouse (B) and fruit fly (C).

The work of Eisenberg highlighted the compactness and short length of human housekeeping genes [13]. We confirmed these results also for the mouse and fruit fly homologs of human housekeeping and tissue specific genes. Figure 1 shows the frequency distribution, normalized to one, of the different cDNA lengths found in housekeeping (HK), tissue specific (TS) and the full gene set, for each of the three species. The housekeeping distribution appears to be shifted to the left of the diagram, reflecting shorter transcripts, while the tissue specific distribution is more dispersed toward the right, reflecting longer exons and introns. Similar patterns of results were found for CDS length and number of exons as summarized in Table 1.

#### Chromatin compactness upstream housekeeping genes

S/MAR are expected to be less present upstream of human housekeeping genes [15], as less packed chromatin eases gene expression. This was confirmed in our analysis (Table 2) also for mouse, while in fruit fly S/MAR sequences seems to be *more *present upstream of housekeeping genes. Poly(dA-dT) sequences, on the contrary, should be more likely present upstream of human housekeeping genes, as they keep the DNA unpackaged and enhance gene expression. This too was confirmed for mouse, while the regions upstream fruit fly genes seems to have almost no Poly(dA-dT) sequences. A pattern similar to that of Poly(dA-dT) sequences is expected for the nucleosomes destabilizing sequence (CCGNN)_2–5_, with increased presence upstream of housekeeping genes to enhance transcription and decreased presence upstream of tissue specific genes [15]. This pattern was confirmed for human and mouse (Table 2) while in fruit fly the opposite seems true. This could be caused by a real biological difference in the structure of housekeeping genes in fruit fly compared to human, or it could be an artefact of the homology conversion from human to fruit fly. In this sense, a fruit fly homologous gene might be similar in function to the human gene, but not share the same pattern of expression, and hence have different chromatin structure. An important aspect of the classifier is that it is independent of the biological interpretation of data. In theory, for the use in the classifier, it doesn't matter what the pattern of difference is, as long as it helps in discriminating between the two classes, but in practice, these new data would deserve further biological analysis, as the role of (CCGNN)_2–5 _sequences has been extensively studied in yeast and human only.

#### Gene Ontology term matching

The Gene Ontology (GO) terms [19] linked to each gene were compared to the GO terms specific for the housekeeping or the tissue specific set of genes. A GO term was defined as being specific for housekeeping genes when the term appears *exclusively *in association with one or more housekeeping genes (for example, a term like *nucleus *[GO:0005634] that appears in 24.5% of human housekeeping genes but also in 13.1% of human tissue specific genes was not used for analysis). The analogous choice was made when generating a list of GO terms specific for tissue specific genes. Table 3 lists the GO terms that appears more frequently in each set. A measure of similarity was generated comparing the GO terms of each gene with the list of GO terms specific for housekeeping (or tissue specific) genes (see the Methods section for a more detailed description).

### Training and evaluation

For the classification of housekeeping genes, only genes classified as housekeeping in all three microarray sets (from Table 4) were accepted in the training set, and only tissue specific genes present in *at least two *of the tissue specific lists from Table 4 were used (please see the Methods section for additional information). A Zero Rule classifier that simply chooses the majority class (here the tissue specific class) as classification for all instances was trained and used as a performance baseline (Table 5). For the actual classification two different Naive Bayes algorithms were tested, a classic version and the AODE (Averaged One-Dependence Estimators) version [20,21] that should reduce the error due to non-independent attributes. Since the performance of two algorithms was very similar (data not shown), from now on we will only comment on the classic Naive Bayes results (Table 6).

**Figure 2:**
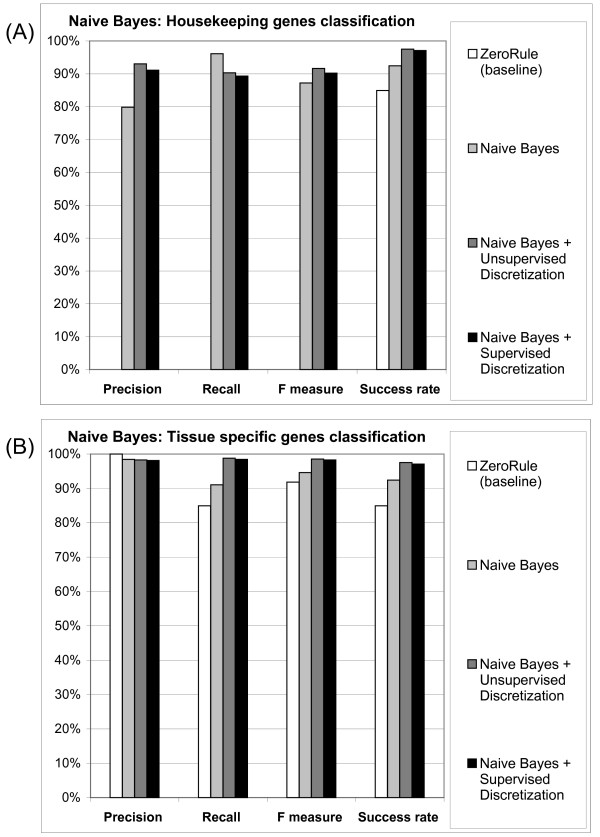
**Effects of discretisation on Naive Bayes classifier performance (human data)**. The success rate value is plotted in both the housekeeping (A) and the tissue specific (B) chart for comparison. Precision, Recall and F Measure for the ZeroRule classifier on housekeeping data (white bar) is equal to zero and hence not directly visible in the housekeeping chart (A).

After data filtering, each classifier was trained and cross-validated for 10-times with a 10-fold random sampling. The ten resulting values for each performance parameter (see the Methods section for the full evaluation procedure) were averaged to obtain the final figures, and Receiver Operating Characteristic (ROC) curves, plotting TP rates vs. FP rates, were analyzed. As expected, the Naive Bayes classifier shows a definite progression in performance when data discretisation is used, either supervised or un-supervised with frequency binning, as shown in Figure 2 for human data. In addition, the performance level is not particularly affected by the threshold of homology chosen. To convert the training set from human to mouse (and fruit fly) thresholds of identity of 40% or 50% have been tested with no significant difference in performance (data not shown).

### Comparing performance across human, mouse and fruit fly

For all species, the classifier performance improves when comparing the ZeroRule classifier baseline with the Naive Bayes classifier (see the Methods section for the definition of performance parameters). In particular, the classification performance over housekeeping genes improves dramatically compared with the 0% Precision, Recall and F Measure of the ZeroRule classifier (Tables 5 and 6).

**Figure 3:**
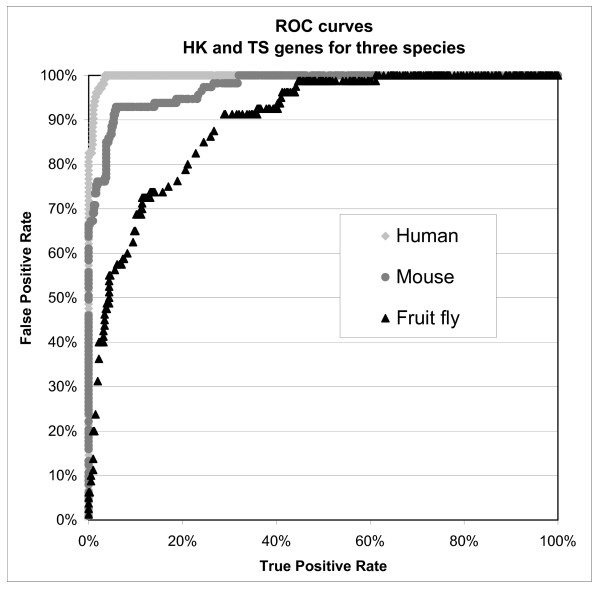
**ROC curves of maximum classification performance for human, mouse and fruit fly**. For human and fruit fly: unsupervised discretisation (with equal frequency binning) + AODE algorithm for classification; for mouse: supervised discretisation + classic Naive Bayes algorithm for classification.

**Figure 4:**
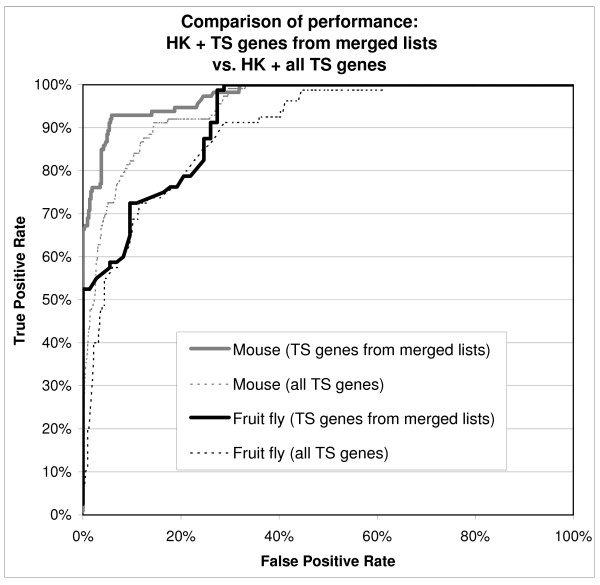
**ROC curves of classification performance: housekeeping + all tissue specific genes versus housekeeping + tissue specific genes from merged lists**. ROC curves comparing the classifier performance when all tissue specific genes or just tissue specific genes present in at least two published lists are used. For human and fruit fly: unsupervised discretisation (with equal frequency binning) + AODE algorithm for classification; for mouse: supervised discretisation + classic Naive Bayes algorithm for classification.

The success rates for the three species are summarized in Table 7. In addition, the Receiver Operating Characteristic (ROC) curves in Figure 3 compare the maximum performance obtained by the classifiers on human, mouse and fruit fly transcripts. ROC curves represent the classification data in a ranked order of probability, showing how many true positives (real housekeeping genes or real tissue specific genes) one can obtain taking, for example, the highest 10% of the ranking list, and how many false positives one would also unknowingly collect in the meantime. Put simply, the closer the curve is to the upper left corner, the better the classification is, while the more the curve relaxes to the right and toward the centre of the diagram, the worse the performance (and the number of false positives collected). The curves in Figure 3 show a decrease in performance for mouse data and, in particular, for fruit fly data when compared to performance on human data. The overall performance was examined further and it seemed unrelated to the identity thresholds, but very sensitive to the data consolidation used. The sensitivity to data consolidation is further analysed in Figure 4: this diagram shows the decrease in performance that appears in both mouse and fruit fly when *all *tissue specific genes are used to generate the training set instead of using only genes present in at least two different published sources.

In general, considering both algorithms (Classic and AODE Naive Bayes) and both filters (supervised and unsupervised) the best success rate that the system could achieve for human is 97 % (all methods), for mouse is 93 % (supervised filtering + Classic Naive Bayes) and for fruit fly is 90 % (unsupervised filtering + AODE) (Table 7).

### Comparison with other classifiers

The Naive Bayes classifier performs better than the baseline on all performance indicators, and was also compared with other well known classifiers. The Naive Bayes classifier is among the best classifiers and learning methods when the success rate is used as main indicator. Interestingly, two of the closest contending algorithms are also derived from the Naive Bayes algorithm (the NB Tree: Naive Bayes Tree and the LBR: Lazy Bayesian Rules Classifier) [22]. The Naive Bayes algorithm is also extremely fast compared to the others tested: there is no significant wait time for a 10-times 10-fold cross-evaluation of ≈ 600 transcripts training set, and it takes only a couple of seconds to obtain the classification of a 40000 instances test set (data not shown).

### Genes and transcripts

In this work, we use transcripts as minimal genetic units for classification, instead of genes. This decision is justified by the biological existence, especially for mammalians, of multiple transcripts for each gene, each with a potentially different tissue expression pattern. In fact, in this study 514 human (2132 mouse, 232 fruit fly) genes having two or more different transcripts had their transcripts classified with different housekeeping probabilities. And 6 human (17 mouse, 15 fruit fly) genes had at least one transcript classified as housekeeping and at least another transcript classified as tissue specific.

The different classification of transcripts of the same gene reflects the ability of the classifier to identify different biological ways of regulating gene expression: by gene length or by chromatin compactness. If a gene contains several transcripts with different patterns of expression, the evolutionary pressure on the chromatin compactness around the gene may be conflicting. The preference for having open chromatin around a housekeeping gene may be some how offset by the pressure for having compact chromatin around a tissue specific transcript. However, there will still be pressure on gene length to keep transcripts often expressed shorter. Similarly, in the classifier, the set of different transcripts for a gene will have all the values for the chromatin attributes in common, as the 3' and 5' regions are the same for all transcripts in a gene. But the classifier can use the other attributes (transcript length and number of exons) to drive the final classification.

### Predicted housekeeping transcripts

In the evaluation phase the classifier was trained on nine tenths of the known data while the remaining tenth was withhold for calculating the classifier performance. In the second phase each classifier was trained with all available known data for each species. In the third phase each of the classifiers was run on all available transcript data for all genes (with known and unknown housekeeping status) to obtain predictions (see additional files 1, 2 and 3). The classifier trained on human data was applied to all 45921 human transcripts (34270 putative genes), the mouse classifier was applied to all 31535 mouse transcripts (24461 putative genes), and the Drosophila classifier was applied to the 20016 Drosophila transcripts (14399 putative genes). Excluding the genes already known to be housekeeping or tissue specific, the three classifiers extracted new lists of housekeeping and tissue specific genes for each of the three species.

The probabilistic classifications uses "housekeeping" and "tissue specific" as the two ends of the spectrum considered. The probability of being housekeeping and being tissue specific add up to one, for example: if the housekeeping probability is 96.5 %, then the tissue specific probability is 3.5%. The actual classification decision will depend on the threshold used. If we use 90% as minimum threshold, all genes with a housekeeping probability above 90% (and hence tissue specific probability below 10%) will be considered housekeeping. Accordingly, all transcripts with tissue specific probability above 90% (and hence housekeeping probability below 10%) will be considered tissue specific.

The number of transcripts with housekeeping probability above 90% and above 50% (a more relaxed threshold) can be found in Table 8. The lists of new housekeeping transcripts found in all three species show a high presence of proteins from housekeeping families, like ribosomal proteins: the related GO terms appear 704 times in the human housekeeping list, 9 times only in the tissue specific predicted set. The classifier also correctly classified as housekeeping mRNAs commonly used as standardization controls like: beta-actin (ACTB), beta-2-microglobulin (B2M), non-POU domain containing nuclear RNA-binding protein (NONO), and the ribosomal proteins RPS27, RPL19, RPL11 and RPS3.

### Predicted tissue specific genes

The number of transcripts with predicted tissue specific probability above 90% (and hence housekeeping probability below 10 %) and tissue specific probability above 50% (housekeeping probability below 50%) are also shown in Table 8. The new predicted tissue specific genes have an extremely varied set of functions, rich in tissue specific structures like brain receptors, muscle fibre components, and also protein variants known to be involved in disease and tumorigenesis. For example, in human, 1133 transcripts have brain specific description (only 16 in the housekeeping set). Some functions are almost completely absent in the housekeeping set, but present in the tissue specific set like: 304 synaptic transmission transcripts (1 in the HK set), 201 spermatogenesis transcripts (1 in the HK set) 221 olfactory receptors transcripts (0 in the HK set), 62 cytokines (0 in the HK set), 32 platelet transcripts (0 in the HK set), 28 GABA transporters and receptors (0 in the HK set), 27 dystrophy involved transcripts (0 in the HK set). The tissue specific set is also particularly enriched in functions like signal transduction (the related GO terms are associated to tissue specific transcripts 1901 times, while only 28 times in the HK set) or receptor activity (GO terms appearing 1553 times in the tissue specific set, but only 12 times in the housekeeping set).

Many other tissue specific functions are represented, like the 5-aminolevulinate synthase erythroid-specific, or involved in disease, like the Abnormal spindle-like microcephaly-associated protein. Other differences between the sets are less quantitatively evident, but not less interesting. For example, among the predicted housekeeping transcripts we find 136 general and mitochondrial elongation factors. In the tissue specific set too we find 96 elongation factors, but not mitochondrial ones, and 36 are testis specific factors, while 16 are negative elongation factors, involved in diseases that subvert the common housekeeping of cell growth, like breast cancer and Wolf-Hirschhorn syndrome, a multiple malformation syndrome characterized by mental and developmental defects.

### Patterns of expression across tissues

**Figure 5:**
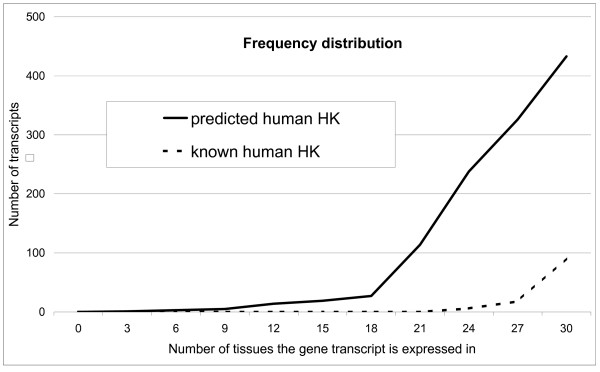
**Tissue expression for known and predicted human housekeeping genes**. Data extracted from UniGene dbEST in July 2005 (UniGene human build 186). The probability for predicted housekeeping transcripts is ≥ 90%. Discretisation: unsupervised; classification algorithm: AODE Naive Bayes.

The pattern of expression across different tissues of the new housekeeping and tissue specific genes can be used to check and confirm the classifier predictions. The UniGene site offers text reports containing the number of EST (Expressed Sequence Tag) found for each UniGene cluster (gene) in 30 basic tissues. The number of EST registered in the database is considered a measure of the level of gene expression in each particular tissue. Figure 5 shows the known and the predicted housekeeping genes, plotted against the number of tissues in which they are expressed. Most of the new predicted human housekeeping genes are expressed in more than 20 tissues out of 30, as would be expected for potential housekeeping genes.

The area between the line representing predicted housekeeping genes (the continuous line above in Figure 5) and the line representing the known transcripts (the dashed line below) visually represents the number of new housekeeping transcripts extracted for human (1251 new transcripts, corresponding to 927 new genes). Since the original lists of housekeeping genes were mainly extracted at a time when Affymetrix microarrays contained only 7000 human genes (against a total of around 25000 today), it would be expected that the classifier would find a number of new housekeeping genes.

## Discussion

The three species investigated (human, mouse and fruit fly) were chosen to generate a certain evolutionary fan, and to test the range of applicability of the method. All theories regarding gene length and chromatin compactness have been verified only in human (and to a limited degree in mouse), but not in fruit fly. However, eukaryotic genes are known to share similar structures and patterns, which have been used in the past to automate gene discovery.

The approach used here is to perform computational experiments, without prior expectations regarding the patterns of mouse or fruit fly data. The results obtained are clearly not random, and the classification performance is good. Therefore, these computational studies broadly confirm that the characteristics of human housekeeping genes can be identified in other species. But the differences between species that we observe also suggest further investigation into characteristic features of such genes for species more distantly related to human, and into the method of determining homologs would indeed be worthwhile.

In particular, one of the main challenges for the training phase was the conversion of the housekeeping and tissue specific genes identifiers: up to 25% of the UniGene gene identifiers published in past works on housekeeping genes were retired from the UniGene database in the last four years. 10% of the UniGene identifiers have been retired without having a new identifier assigned, and so part of the potential housekeeping training set was lost. The identifiers conversion between the NCBI accession numbers (or Entrez genes identifiers) and the European EMBL identifiers caused a loss of around 15% of the genes.

In addition, the available tissue specific lists agreed on just 7 transcripts out of 5225 human transcripts from [7], 5740 from [23] and 680 from [6], (but the authors agree on the actual tissue the gene is expressed in only for one transcript). However, to create a strong training set and to preserve high performance we still tried to merge the available list to obtain only the data on which different authors and different experimental techniques agreed. Another constraint was the ratio between housekeeping and tissue specific genes: from microarray estimates in *Homo sapiens *housekeeping genes represent about 5–7 % of the total [7], but there is no evidence that the human estimate applies also to the other species.

## Conclusion

To-date, studies of housekeeping genes have typically concentrated on human genes only, and the housekeeping genes are normally taken from a small number of already published lists. The labelling of genes with housekeeping or tissue specific status is not common, even in highly curated databases. In this work, we propose an automated solution that is based on the integration of existing datasets. As noted above, integration is not straightforward, but is achievable.

The main achievement of this work is to have proved that it is possible to discover if a gene is housekeeping using simple features of that gene sequence and its surroundings. This is made possible by the integration of different attributes operated by the Naive Bayes engine, since the attributes already discovered in literature (like the association between exon length and being an housekeeping gene) were not powerful enough, taken alone, for deciding if a gene was housekeeping or not. This opens up the possibility of automatically assigning the housekeeping/tissue specific status to all genes (or potential ORFs) for which we have a sequence.

Our method proposes not only a housekeeping label, but a certainty value, that gives a measure of trust in the prediction. This classification method is applicable to genes of any eukaryotic species, exploiting information that is already available in publicly accessible databases, and it can generate a functional description even for sequenced but otherwise unknown genes. Considering this strength of the method here evaluated, future work might include the analysis of less well known genomes.

At the same time, in the future the structure of the classifier might be enriched, for example, the Gene Ontology structure might be further exploited to compare also GO terms that are in a parent or child relation with the GO terms of each gene. As the risk of overfitting is always present with attributes that are partially learnt from the training set, the GO attributes could be further elaborated, for example: using housekeeping and tissue specific lists of GO terms verified by expert curators, or by learning the GO lists from other sources. Thanks to the flexible structure of the Naive Bayes classifier additional attributes can be easily added, either attributes already studied (for example, the Alu repeats for chromatin compactness already analyzed in [15]) or newly discovered ones.

## Methods

### Software

The EMBOSS suite [24,25] program *MARsearch *was used to extract the presence of S/MARs and the *dreg *program to extract the Poly(dA-dT) and (CCGNN)n sequences present in the 5' and 3' regions of each transcript. The Weka Data Mining Java suite [26,27] was used for training and testing the Naive Bayes classifier and for the comparison to other learning algorithms. A Python script was used to extract the number of tissues each gene is expressed in from the reports available at the UniGene FTP site [28]. A Microsoft Access database (Office 2003) was used for storing and manipulating all the data. The database was accessed through the proprietary interface, and from Java with a JDBC-ODBC bridge.

### Algorithms

#### Discretisation

The Weka algorithm used for filtering with Unsupervised discretisation involves separating the data in ranges using equal-frequency binning (histogram equalization) so that the same number of training example fall into each bin. No class information is taken into consideration [29]. For Supervised discretisation the data is separated in intervals as homogeneous as possible in relation to class content. The entropy based method with MDL (Minimum Description Length) stopping criterion [30] is used to define where to stop when segmenting the intervals.

#### Classifiers

All the algorithms used were taken from the Weka suite [26,27]. In addition to the classic Naive Bayes algorithm and the AODE (Averaged One-Dependence Estimators) version [20,21], the other classifiers used were: Adaboost Ml method, Alternating Decision Tree (AD Tree), Decision Table, J48 (a variant of the C4.5 decision tree), Lazy Bayesian Rules Classifier (LBR), Logistic Model Trees (LMT), Naive Bayes tree (NB Tree), One Rule classifier (1R classifier), PART decision list, Ridge Logistic Regression and RIpple-DOwn Rule learner (Ridor). For a comparison of performance see [22].

### Evaluation

All evaluation parameters are calculated with a ten times, ten fold cross-evaluation. The method uses nine tenths of the data for training the system while the remaining tenth is set aside as a test set (control) for estimating the various evaluation parameters, like the success rate (see below for parameters definition). The data is randomised and the procedure is repeated 10 times to estimate the average value for each parameter. The parameters used for evaluation are the following (where TP = true positive, FP = false positive, TN = true negative and FN = false negative). Precision: defined as the number of positive instances retrieved over the total number of instances declared positive by the classifier (= TPTP+FP
 MathType@MTEF@5@5@+=feaafiart1ev1aaatCvAUfKttLearuWrP9MDH5MBPbIqV92AaeXatLxBI9gBaebbnrfifHhDYfgasaacH8akY=wiFfYdH8Gipec8Eeeu0xXdbba9frFj0=OqFfea0dXdd9vqai=hGuQ8kuc9pgc9s8qqaq=dirpe0xb9q8qiLsFr0=vr0=vr0dc8meaabaqaciaacaGaaeqabaqabeGadaaakeaadaWcaaqaaiabdsfaujabdcfaqbqaaiabdsfaujabdcfaqjabgUcaRiabdAeagjabdcfaqbaaaaa@3490@) Recall: defined as the number of true positive instances retrieved over the total number of instances that are positive in the set (= TPTP+FN
 MathType@MTEF@5@5@+=feaafiart1ev1aaatCvAUfKttLearuWrP9MDH5MBPbIqV92AaeXatLxBI9gBaebbnrfifHhDYfgasaacH8akY=wiFfYdH8Gipec8Eeeu0xXdbba9frFj0=OqFfea0dXdd9vqai=hGuQ8kuc9pgc9s8qqaq=dirpe0xb9q8qiLsFr0=vr0=vr0dc8meaabaqaciaacaGaaeqabaqabeGadaaakeaadaWcaaqaaiabdsfaujabdcfaqbqaaiabdsfaujabdcfaqjabgUcaRiabdAeagjabd6eaobaaaaa@348C@); F Measure: combines precision and recall (= 2 Recall PrecisionRecall+Precision
 MathType@MTEF@5@5@+=feaafiart1ev1aaatCvAUfKttLearuWrP9MDH5MBPbIqV92AaeXatLxBI9gBaebbnrfifHhDYfgasaacH8akY=wiFfYdH8Gipec8Eeeu0xXdbba9frFj0=OqFfea0dXdd9vqai=hGuQ8kuc9pgc9s8qqaq=dirpe0xb9q8qiLsFr0=vr0=vr0dc8meaabaqaciaacaGaaeqabaqabeGadaaakeaadaWcaaqaaiabikdaYiabbccaGGqaciab=jfasjab=vgaLjabdogaJjabdggaHjabdYgaSjabdYgaSjabbccaGiab=bfaqjab=jhaYjabdwgaLjabdogaJjabdMgaPjabdohaZjabdMgaPjabd+gaVjabd6gaUbqaaiab=jfasjab=vgaLjabdogaJjabdggaHjabdYgaSjabdYgaSjabgUcaRiab=bfaqjab=jhaYjabdwgaLjabdogaJjabdMgaPjabdohaZjabdMgaPjabd+gaVjabd6gaUbaaaaa@5813@, or: 2TP2TP+FP+FN
 MathType@MTEF@5@5@+=feaafiart1ev1aaatCvAUfKttLearuWrP9MDH5MBPbIqV92AaeXatLxBI9gBaebbnrfifHhDYfgasaacH8akY=wiFfYdH8Gipec8Eeeu0xXdbba9frFj0=OqFfea0dXdd9vqai=hGuQ8kuc9pgc9s8qqaq=dirpe0xb9q8qiLsFr0=vr0=vr0dc8meaabaqaciaacaGaaeqabaqabeGadaaakeaadaWcaaqaaiabikdaYiabdsfaujabdcfaqbqaaiabikdaYiabdsfaujabdcfaqjabgUcaRiabdAeagjabdcfaqjabgUcaRiabdAeagjabd6eaobaaaaa@3990@); Success Rate: the number of real positive and negative instances retrieved over the total number of instances (= TP+TNTP+TN+FP+FN
 MathType@MTEF@5@5@+=feaafiart1ev1aaatCvAUfKttLearuWrP9MDH5MBPbIqV92AaeXatLxBI9gBaebbnrfifHhDYfgasaacH8akY=wiFfYdH8Gipec8Eeeu0xXdbba9frFj0=OqFfea0dXdd9vqai=hGuQ8kuc9pgc9s8qqaq=dirpe0xb9q8qiLsFr0=vr0=vr0dc8meaabaqaciaacaGaaeqabaqabeGadaaakeaadaWcaaqaaiabdsfaujabdcfaqjabgUcaRiabdsfaujabd6eaobqaaiabdsfaujabdcfaqjabgUcaRiabdsfaujabd6eaojabgUcaRiabdAeagjabdcfaqjabgUcaRiabdAeagjabd6eaobaaaaa@3E1C@); Root Mean Squared Error: (p1−a1)2+...+(pn−an)2n
 MathType@MTEF@5@5@+=feaafiart1ev1aaatCvAUfKttLearuWrP9MDH5MBPbIqV92AaeXatLxBI9gBaebbnrfifHhDYfgasaacH8akY=wiFfYdH8Gipec8Eeeu0xXdbba9frFj0=OqFfea0dXdd9vqai=hGuQ8kuc9pgc9s8qqaq=dirpe0xb9q8qiLsFr0=vr0=vr0dc8meaabaqaciaacaGaaeqabaqabeGadaaakeaadaGcaaqaamaalaaabaGaeiikaGIaemiCaa3aaSbaaSqaaiabigdaXaqabaGccqGHsislcqWGHbqydaWgaaWcbaGaeGymaedabeaakiabcMcaPmaaCaaaleqabaGaeGOmaidaaOGaey4kaSIaeiOla4IaeiOla4IaeiOla4Iaey4kaSIaeiikaGIaemiCaa3aaSbaaSqaaiabd6gaUbqabaGccqGHsislcqWGHbqydaWgaaWcbaGaemOBa4gabeaakiabcMcaPmaaCaaaleqabaGaeGOmaidaaaGcbaGaemOBa4gaaaWcbeaaaaa@4526@, where *p*_1_, *p*_2_,..., *p*_*n *_are the predicted values for each transcript, *a*_1_, *a*_2_,..., *a*_*n *_are the actual values and n is the total number of predictions (number of transcripts considered). The standard deviation over the ten success rate values and over the ten root mean squared error values is calculated as follows

∑i=0N(xi−x¯)2N−1.
 MathType@MTEF@5@5@+=feaafiart1ev1aaatCvAUfKttLearuWrP9MDH5MBPbIqV92AaeXatLxBI9gBaebbnrfifHhDYfgasaacH8akY=wiFfYdH8Gipec8Eeeu0xXdbba9frFj0=OqFfea0dXdd9vqai=hGuQ8kuc9pgc9s8qqaq=dirpe0xb9q8qiLsFr0=vr0=vr0dc8meaabaqaciaacaGaaeqabaqabeGadaaakeaadaGcaaqaamaalaaabaWaaabCaeaacqGGOaakcqWG4baEdaWgaaWcbaGaemyAaKgabeaakiabgkHiTiqbdIha4zaaraGaeiykaKYaaWbaaSqabeaacqaIYaGmaaaabaGaemyAaKMaeyypa0JaeGimaadabaGaemOta4eaniabggHiLdaakeaacqWGobGtcqGHsislcqaIXaqmaaaaleqaaOGaeiOla4caaa@3FD5@

### Data

In this work we used the EMBL [31,32] database version Ensembl 34, based on the following assemblies. For human: NCBI 35 assembly (July 2004). For mouse: NCBI m34 mouse assembly (freeze May 17, 2005, strain C57BL/6J). For fruit fly: BDGP 4 assembly (Apr 2005), FlyBase gene build (Feb 2005). The length and sequence data, and the GO terms were extracted from the EMBL Genome Browser using the EnsMart (now BioMart) batch query interface [31]. The 5' UTR region analyzed for chromatin compactness signals corresponds to the 1500 bp upstream the transcription start (for the 3' UTR region, the 1500 bp downstream the translation stop were analyzed). Data from the EMBL Ensembl was also used for cross-species homology. In Table 4 we summarized the author, data provenance, number of genes analyzed and technique used for each of the published lists used. The UniGene identifiers history files were downloaded from the UniGene web site [33]. Data regarding the pattern of expression in different tissues were extracted with a Python script from the UniGene reports [34] based on the dbEST version of July 2005 (the UniGene build was 186 for human and 148 for mouse; fruit fly reports not available).

### Training sets

For the classification of human genes: only housekeeping genes present in all three list were accepted in the training set, and only tissue specific genes present in *at least two *of the tissue specific lists from Table 4 were used. The same criteria were used for mouse and fruit fly. In addition, only pairs of human/mouse (or human/fruit fly) transcripts that surpassed identity and coverage thresholds of 50% were accepted as being homologous to generate the mouse (or fruit fly) training set. This procedure created a set of around 100 housekeeping transcripts for each species (specifically: 76 genes/103 transcripts for human, 93 genes/113 transcripts mouse and 40 genes/80 transcripts fruit fly). For tissue specific genes a full merging would have been too limiting, as only a handful of genes are present in all lists. Therefore we resorted to include all tissue specific genes that were supported by *at least two *independent experiments, collecting a set of 326 genes/580 transcripts for human and 286 genes/564 transcripts mouse. For fruit fly, however, the homology conversion from human had already heavily reduced the number of usable genes and, if tissue specific genes from at least two lists are used, at the end of the merging the percentage of housekeeping genes is near 50% and the number of genes only 74. The alternative chosen was to accept *all *tissue specific genes available, bringing the ratio back to the human level and the gene number up 193/412 transcripts, even if this leads to a degradation in performance as shown in Figure 4.

### Attributes

To generate the percent of matching with specific Gene Ontology (GO) terms for each transcript, two preliminary lists of terms were generated: the *Housekeeping GO terms list*, which contains the identifiers of all GO terms connected to housekeeping genes (terms also connected to any tissue specific gene were excluded from the list), and the *Tissue specific GO terms list*: a list containing the identifiers of all GO terms connected to tissue specific genes (and not connected to any housekeeping gene). The percent of matching with these lists was then calculated for each gene. For example, if a gene is annotated with a total of 5 GO terms, of which 3 are present in the housekeeping GO terms list, and 1 is in the tissue specific GO terms list, the percentages will be: 60% of matching for the housekeeping GO terms and 20% of matching for the tissue specific GO terms.

## Authors' contributions

LDF designed and implemented the classifier, designed the study, and carried out the experiments. SA assisted with the design of the study and helped to draft the manuscript. Both authors read and approved the final manuscript.

## Supplementary Material

Additional file 1 **Attributes values and housekeeping probabilities for all EMBL *human *genes**. The file contains the following attributes in tab separated format: 1. EMBL_gene_id = The EMBL gene identifier, 2. HGNC_symbol = the HUGO Gene Name Committee identifier 3. description = a textual description of the gene function, 4. EMBL_transcript_id = The EMBL transcript identifier, 5. cDNA_length = cDNA length (entire pre-splicing mRNA length: exons + introns + other untranslated regions), 6. cds_length = Coding sequence length (exons only), 7. exons_nr = Number of exons, 8. 3_MAR_presence = Presence of S/MAR in the 3' region, 9. 5_MAR_presence = Presence of S/MAR in the 5' region, 10. 5_polyA_18_presence = Presence of Poly(dA-dT) (with length of 18 or more bp) in the 5' region, 11. 5_CCGNN_2_5_presence = Presence of (CCGNN)_2–5 _in the 5' region, 12. perc_go_ts_match = Percent of GO terms for the gene that match with the tissue specific GO terms list, 13. perc_go_hk_match = Percent of GO terms for the gene that match with the housekeeping GO terms list, 14. is_hk = The housekeeping or tissue specific former classification from published lists (when known), 15. predicted_class = The predicted class given the probability (class is housekeeping if housekeeping probability ≥ 50%, tissue specific if probability ≤ 50%) 16. hk_probability = The new housekeeping probability generated by the Naive Bayes classifier When a value was unknown it was represented by a question mark, following the "arff" file standard for machine learning. Click here for file

Additional file 2 **Attributes values and housekeeping probabilities for all EMBL *mouse *genes**. The file contains the following attributes in tab separated format: 1. EMBL_gene_id = The EMBL gene identifier, 2. MGI_symbol = the Mouse Genomic Informatics (MGI) symbol 3. description = a textual description of the gene function, 4. EMBL_transcript_id = The EMBL transcript identifier, 5. cDNA_length = cDNA length (entire pre-splicing mRNA length: exons + introns + other untranslated regions), 6. cds_length = Coding sequence length (exons only), 7. exons_nr = Number of exons, 8. 3_MAR_presence = Presence of S/MAR in the 3' region, 9. 5_MAR_presence = Presence of S/MAR in the 5' region, 10. 5_polyA_18_presence = Presence of Poly(dA-dT) (with length of 18 or more bp) in the 5' region, 11. 5_CCGNN_2_5_presence = Presence of (CCGNN)_2–5 _in the 5' region, 12. perc_go_ts_match = Percent of GO terms for the gene that match with the tissue specific GO terms list, 13. perc_go_hk_match = Percent of GO terms for the gene that match with the housekeeping GO terms list, 14. is_hk = The housekeeping or tissue specific former classification from published lists (when known), 15. predicted_class = The predicted class given the probability (class is housekeeping if housekeeping probability ≥ 50%, tissue specific if probability ≤ 50%) 16. hk_probability = The new housekeeping probability generated by the Naive Bayes classifier When a value was unknown it was represented by a question mark, following the "arff" file standard for machine learning. Click here for file

Additional file 3 **Attributes values and housekeeping probabilities for all EMBL *fruit fly *genes**. The file contains the following attributes in tab separated format: 1. EMBL_gene_id = The EMBL gene identifier, 2. FlyBase_symbol = the FlyBase symbol 3. description = a textual description of the gene function, 4. EMBL_transcript_id = The EMBL transcript identifier, 5. cDNA_length = cDNA length (entire pre-splicing mRNA length: exons + introns + other untranslated regions), 6. cds_length = Coding sequence length (exons only), 7. exons_nr = Number of exons, 8. 3_MAR_presence = Presence of S/MAR in the 3' region, 9. 5_MAR_presence = Presence of S/MAR in the 5' region, 10. 5_polyA_18_presence = Presence of Poly(dA-dT) (with length of 18 or more bp) in the 5' region, 11. 5_CCGNN_2_5_presence = Presence of (CCGNN)_2–5 _in the 5' region, 12. perc_go_ts_match =Percent of GO terms for the gene that match with the tissue specific GO terms list, 13. perc_go_hk_match = Percent of GO terms for the gene that match with the housekeeping GO terms list, 14. is_hk = The housekeeping or tissue specific former classification from published lists (when known), 15. predicted_class = The predicted class given the probability (class is housekeeping if housekeeping probability ≥ 50%, tissue specific if probability ≤ 50%) 16. hk_probability = The new housekeeping probability generated by the Naive Bayes classifier When a value was unknown it was represented by a question mark, following the "arff" file standard for machine learning. Click here for file

## Figures and Tables

**Table 1:** Difference in length and exon number between housekeeping and tissue specific transcripts.

	Human			Mouse			Fruit fly		
	HK	TS	P value	HK	TS	P value	HK	TS	P value

Average cDNA length (bp)	1681	2534	l.0E-09	1271	2402	5.6E-17	1413	2705	2.8E-18
Average CDS length (bp)	899	1670	5.9E-16	817	1623	2.8E-18	864	1886	2.5E-18
Average number of exons	7.2	10.7	6.0E-04	6.7	11.4	4.2E-13	4.0	7.3	1.7E-21

HK = housekeeping, TS = tissue specific. P value for t-test two tail hypothesis.

**Table 2:** Presence of sequences involved in chromatin packaging in housekeeping and tissue specific genes.

	Human			Mouse			Fruit fly		
	All genes (%)	HK (%)	TS (%)	All genes (%)	HK (%)	TS (%)	All genes (%)	HK (%)	TS (%)

5' S/MAR	11	5	9	9	3	7	22	16	24
3' S/MAR	13	7	13	10	7	10	21	20	26
5' Poly(dA-dT) ≥ 12 bp	30	31	29	22	28	19	10	10	8
5' Poly(dA-dT) ≥ 15 bp	21	24	20	14	25	13	4	1	0
5' Poly(dA-dT) ≥ 18 bp	13	17	11	8	20	7	1	1	0
5' Poly(dA-dT) ≥ 20 bp	10	13	7	7	10	6	1	1	0
5' (CCGNN)2–5	22	47	20	19	27	12	15	10	16

5' = the sequence is present in the 1500 bp upstream the transcription start, 3' = the sequence is present in the 1500 bp downstream the transcription end, HK = housekeeping, TS = tissue specific.

**Table 3:** GO terms specific for the human housekeeping or tissue specific genes set

GO terms specific for:	GO ID	Description	% of HK genes	% of TS genes
HK genes	GO:0006412	protein biosynthesis	29.1	-
	GO:0003735	structural constituent of ribosome	23.3	-
	GO:0005840	ribosome	18.4	-
	GO:0005842	cytosolic large ribos. subunit (sensu Euk.)	10.7	-
	GO:0030529	ribonucleoprotein complex	4.9	-
	GO:0006414	translational elongation	3.9	-

TS genes	GO:0005615	extracellular space	-	14.8
	GO:0004295	trypsin activity	-	8.4
	GO:0004263	chymotrypsin activity	-	8.4
	GO:0005576	extracellular region	-	6.7
	GO:0006810	transport	-	6.6
	GO:0008233	peptidase activity	-	6.6

GO = Gene Ontology, GO ID = Gene Ontology term identifier, HK = housekeeping, TS = tissue specific.

**Table 4:** Published lists of housekeeping and tissue specific genes

Author, Year	Data originally from	Type	Nr. of genes extracted	Organism	Technique	Nr. of tissues	Genes assayed
Eisemberg, 2003 [13]	Su, 2002 [8]	HK	575	Human	Affymetrix ma	25	n/a
Warrington, 2000 [6]	Warrington, 2000 [6]	HK	535	Human	Affymetrix ma	11	about 7000
Haverty, 2002 [35]	Hsiao, 2001 [7]	HK	451	Human	Affymetrix ma	19	about 7000
Haverty, 2002 [35]	Hsiao, 2001 [7]	TS	1525	Human	Affymetrix ma	19	about 7000
Ge, 2005 [23]	Ge, 2005 [23]	TS	1687	Human	Affymetrix ma	33	about 20000
Warrington, 2000 [6]	Warrington, 2000 [6]	TS	170	Human	Affymetrix ma	11	about 7000

HK = housekeeping, TS = tissue specific,, n/a = not available, Affymetrix ma = microarray from Affymetrix (Santa Clara, CA). All tissues are normal adult tissues.

**Table 5:** Zero Rule classifier performance

		Precision (%)	Recall (%)	F Measure (%)	Success Rate (%)	Root Mean Squared Error
Human	HK	0.0	0.0	0.0	84.92 ± 0.01	0.36 ± 0.01
	TS	100.0	84.9	91.8		
Mouse	HK	0.0	0.0	0.0	79.12 ± 0.01	0.41 ± 0.01
	TS	100.0	79.1	88.3		
Fruit fly	HK	0.0	0.0	0.0	83.74 ± 0.01	0.37 ± 0.01
	TS	100.0	68.7	52.3		

HK = housekeeping, TS = tissue specific, null = not possible to calculate the value (a zero appears as the denominator of a division). See the Methods section for the definition of Precision, Recall, F Measure and Success Rate.

**Table 6:** Naive Bayes classifier performance with unsupervised discretisation

		Precision	Recall	F Measure	Success Rate	Root Mean Squared Error
Human	HK	93.0	90.3	91.6	97.49 ± 0.14	0.13 ± 0.01
	TS	98.3	98.8	98.5		
Mouse	HK	83.3	79.6	81.4	92.57 ± 0.2	0.24 ± 0.01
	TS	94.7	95.8	95.2		
Fruit fly	HK	63.2	60.0	61.5	87.46 ± 0.34	0.32 ± 0.01
	TS	92.3	93.2	92.8		

HK = housekeeping, TS = tissue specific. Discretisation: unsupervised with equal frequency binning. See the Methods section for the definition of Precision, Recall, F Measure and Success Rate.

**Table 7:** Success Rate for ZeroRule and Naive Bayes

	ZeroRule	Naive Bayes	
Discretisation		Unsupervised	Supervised

Human	84.92 ± 0.01	97.49 ± 0.14	97.34 ± 0.1
Mouse	79.12 ± 0.01	92.57 ± 0.2	93.18 ± 0.19
Fruit fly	83.74 ± 0.01	87.46 ± 0.34	86.41 ± 0.23

HK = housekeeping, TS = tissue specific. Discretisation: unsupervised with equal frequency binning. See the Methods section for the definition of Success Rate.

**Table 8:** Number of new transcripts with housekeeping probability

Housekeeping probability	≥ 90%	> 50%	≤ 50%	≤ 10%
Predicted class	HK	HK	TS	TS
Human	1342 (3%)	5570 (12%)	40151 (87%)	20686 (45%)
Mouse	3375 (11%)	8001 (25%)	23534 (75%)	16367 (52%)
Drosophila	287 (1%)	3410 (17%)	16606 (83%)	11780 (59%)

HK = housekeeping, TS = tissue specific. The values between parentheses represent the percentage over the entire set of transcripts for the given species. For human and fruit fly: unsupervised discretisation (with equal frequency binning) + AODE algorithm for classification; for mouse: supervised discretisation + classic Naive Bayes algorithm for classification.
